# Hematological reference intervals determination in adults at Gondar university hospital, Northwest Ethiopia

**DOI:** 10.1186/s13104-016-2288-8

**Published:** 2016-11-02

**Authors:** Aregawi Yalew, Betelihem Terefe, Meseret Alem, Bamlaku Enawgaw

**Affiliations:** 1 Department of Hematology & Immunohematology, School of Biomedical and Laboratory Sciences, College of Medicine and Health Sciences, University of Gondar, P.O. Box 196, Gondar, Ethiopia; 2 Department of Immunology & Molecular Biology, School of Biomedical and Laboratory Sciences, College of Medicine and Health Sciences, University of Gondar, Gondar, Ethiopia

**Keywords:** Hematological reference interval, Adult population, Gondar

## Abstract

**Background:**

Hematological reference values are important for the clinical decisions in laboratory diagnosis and monitoring of patients. The correct interpretation of laboratory results depends entirely on the reference intervals that have been established for the locality. But, in sub-Saharan African countries particularly in Ethiopia, locally derived reference intervals were not established and they are forced to use intervals established from western population. Thus this study aimed to establish locally derived hematological reference values that could be used in Northwest Ethiopia.

**Methods:**

A cross sectional study was conducted from April to May 2014 with 120 male and 120 female apparently health adult blood donors at Gondar University Hospital. A structured pretested questionnaire was used for socio demographic and clinical data collection. About 4 ml of blood was collected with EDTA test tube and analyzed using Cell-Dyn 1800 to enumerate the hematological parameters. The data were collected and entered into SPSS version 20 for analysis. Mann–Whitney U test was used to determine reference intervals and Harris and Boyd test was used to determine the reference intervals that need partition. The 95th percentile of measurements was taken as a reference interval.

**Results:**

Median and 95th percentile of WBC for general population were lower than Caucasian population, Addis Ababa, Burkina Faso and Kenya of similar studies. The RBC, Hgb and PCV lower 95% limit values of both sex were lower than studies in Addis Ababa, Kenya, Burkina Faso and text book. While PCV upper limit values higher than the above countries. MCV values of the current study were higher than those countries while MCHC values were lower. Similarly, the absolute values of neutrophils in the current study were lower than Caucasian and Afro Caribbean but higher than African countries and Jamaica but lymphocyte count was higher.

**Conclusions:**

The hematological reference intervals established in this study was different from those reported in other part of Ethiopia or African countries as well as Caucasian population. The RBC, PCV, Hgb and MCHC reference intervals were different in gender. Thus, using of locally determined reference range is advisable.

**Electronic supplementary material:**

The online version of this article (doi:10.1186/s13104-016-2288-8) contains supplementary material, which is available to authorized users.

## Background

Hematological reference values are necessary in routine assessments for diagnosis of blood disorders, infectious diseases, immune status, and diseases progression and also in assessment of response to antiretroviral treatment. Locally-derived age-specific hematological reference ranges were also recommended for proper patient managements and interpretation of results [1].

In the use and interpretation of laboratory test results, it is important to understand the limitations of tests. These include the ability of the test to indicate the presence or absence of disease or whether the value in a report is normal or abnormal for a patient. These sounds the knowledge of reference value for a particular laboratory test is very crucial in patient management. The laboratory staff and those requesting tests should know the accepted reference ranges and clinical significance of the results of the quantitative tests performed in the laboratory. This will ensure that significantly abnormal results are detected, checked, reported, and acted on as soon as possible [2].

There is a difference in reference ranges related to age, sex, geographic origin, altitude, and ethnic background in addition to hemoglobin abnormalities (thalassemia, sickle cell disease and hemoglobin C) or pathologic conditions (malaria, HIV and other viral infections) prevented to have one common hematological values for all countries [2–7]. Thus, the use of normal hematological values derived from other African countries could result in incorrect patient management in routine clinical care.

The reference intervals for analysis of hematological parameters during routine clinical activities in Ethiopia or Africa are reference texts generated from Caucasian adult populations living in developed countries [8]. Lower values for hemoglobin, red blood cells, hematocrit, mean corpuscular volumes, platelets and neutrophils and higher monocytes and eosinophils levels were reported in African population compared to similar population in Western counter parts [6, 9–12]. Also there are ample evidences that clinicians and medical researchers should use method-specific reference ranges in their laboratories which account for gender differences and variances in the ethnic composition of the local society [13], but this is not done in developing countries.

An increasing number of clinical trials taking place in Africa are seeking to identify safe and effective prevention and treatment strategies to combat the heavy burden of infectious diseases in this region but there is only attempts to establish standard reference hematological intervals [14], there is lack of well-established reference intervals.

In the absence of locally derived reference intervals, clinicians and researchers have to use reference values of western population’s, thus it is important to establish local hematological reference intervals for appropriate diagnosis, treatment, and follow up of patients. The reference intervals that are currently used in Ethiopia are adopted from textbooks that refer mainly to Caucasian subjects. But one study in Ethiopia conducted in southeast Addis Ababa showed lower WBC and platelet values of healthy HIV negative Ethiopians than the adopted reference values [15]. Thus, the main aim of this study was to establish locally derived hematological reference values that could be used in Northwest Ethiopia.

## Methods

### Study setting and population

A cross sectional study was conducted from April to June 2014 at Gondar University Hospital blood banking center. The hospital is located in Gondar town which is 740 km from the capital of Ethiopia, Addis Ababa, in the Northwest of Ethiopia. The city has a latitude and longitude of 12°36′N and 37°28′E with an elevation of 2133 meters above sea level. Based on figures from the Central Statistical Agency of Ethiopia in 2007, Gondar has an estimated total population of 231,977. Gondar University Hospital is the only referral hospital with more than 400 beds for North West Ethiopia serving a population of about 5 million. The consecutive nonparametric method was based solely on the ranks of the observations (in order of magnitude) and ignores their measured values. According to the National Committee for Clinical Laboratory Standards (NCCLS), International Federation of Clinical Chemistry (IFCC) and Clinical Laboratory Standards Institute (CLIS) Guideline recommendations, a minimum size of 120 observations from each category is needed [16]. Based on this guide line 120 male and 120 female study participants who pass the exclusion/inclusion criteria were selected conveniently. The reference individuals were selected based on medical examinations, current health status, blood pressure, taking any medication, working with hazardous chemicals, alcohol intake, presence of inherited health disorder in the family, tuberculosis, lymphadenopathy, weight loss, regular exercise, tobacco smoking, allergy manifestation, fever, malaria, HIV, hepatitis B virus surface antigen (HbsAg), hepatitis C virus antibodies (HcAbs), menstruation, pregnancy and use of contraceptives. After written informed consents were taken from study subjects, 4 ml of venous blood samples were collected immediately following the donation in tube containing EDTA and 4 ml bloods with plan tube for serological analysis was taken and proceed.

### Laboratory investigation

About 4 ml of venous blood was collected by an experienced laboratory technologist from each subject for hematological parameters analysis. Hematological parameters; total white cell count (WBC), differential white cell count (neutrophils, lymphocytes, eosinophils, monocytes and basophiles), platelet count, red blood cell count (RBC), hemoglobin (Hgb), hematocrit (%), mean cell volume (MCV), mean cell hemoglobin (MCH), mean cell hemoglobin concentration (MCHC), and red cell distribution width (RDW) were determined using the automated blood analyzer Cell-Dyne 1800 (Abbott Laboratories Diagnostics Division, USA).The other 4 ml of blood after clotting the serum was separated and serological tests (HIV, HbsAg, and HcAbs) was assayed. Erythrocyte sedimentation rate (ESR) was done by using westerngreen method for 1 h.

### Statistical analysis

Data was cleaned, edited, checked for completeness and entered into Epi-Info version 3.3.5 and then transferred to SPSS version 20 for statistical analysis. The mean, median and standard deviation of hematological reference intervals were determined using descriptive statistics. Mann–Whitney U test was used to determine reference intervals and also Harris and Boyd test was used to determine the reference intervals that need partition (Table 2). The 95th percentile was taken as a reference interval.

## Results

### Socio demographic characteristics

A total of 120 male and 120 female adult healthy subjects from Gondar University Hospital Blood Bank Centre were included in this study. The mean age of the study subjects was 24.50 ± 6.65 years with age range from 18 to 50 years and about 69.6% were within age range of 18–25 years. From the study subjects 78.3% were living in Gondar town, 26.7% were married, 58.3% were students and 89.2% were orthodox in religion (Table 1).

**Table 1:** The socio demographic characteristics of adult of Gondar participated in the study

Variables	Socio demographic characteristics	Frequency	Percentage (%)
Sex	Male	120	50
	Female	120	50
Age group (years)	18–25	167	69.6
	26–35	51	21.2
	>35	22	9.2
Address	Gondar	188	78.9
	Around gondar	52	21.1
Marital status	Married	64	26.7
	Single	174	72.5
	Divorced	2	0.8
Religion	Orthodox	214	89.2
	Muslim	22	9.2
	Protestant	1	0.4
	Others	3	1.2
Occupation	Civil servant	21	8.8
	Private	36	15.0
	House wife	6	2.5
	Farmer	37	15.4
	Students	120	58.3

### Hematological reference interval

In order to determine hematological parameters that need combined or partition reference interval based on sex, Harris and Boyd test was used. Basing this test partitioned reference interval was determined for RBC, PCV, Hgb, MCH and MCHC (Table 2). Median and 95th percentile of WBC, absolute neutrophils, absolute lymphocytes, absolute mixed cell, platelet, MCV, RDW and ESR were 5.1 (3.2–8.8 × 10^9^/l), 2.7 (1.6–5.1 × 10^9^/l), 1.9 (1–3.5 × 10^9^/l), 0.5 (0.2–1 × 10^9^/l), 264 (128–432 × 10^9^/l), 92 (85–100 fl); 14 (12–17%) and 5 (0–15 mm/h) respectively for the general population (Table 3).

**Table 2:** Result of Harris and Boyd test, which was performed to see the need for partitioning of Reference interval based on sex

Parameters	P value	Harris and Boyd		
		Z*	Z^†^	Decision
WBC (×10^9^/l)	0.3779	3	0.4	No separate RI
Neutrophil (%)	0.7239	3	0.6	No separate RI
Neutrophils absolute (×10^9^/l)	0.6331	3	0.01	No separate RI
Lymphocyte (%)	0.6750	3	0.5	No separate RI
Lymphocytes absolute (×109/l)	0.1705	3	0.9	No separate RI
Mixed (%)	0.5375	3	0.5	No separate RI
Mixed absolute (×10^9^/l)	0.2784	3	0.5	No separate RI
Platelet (×10^9^/l)	0.0223	3	2.3	No separate RI
RBC (×10^12^/l)	*0.0016*	3	3.4	Separate RI
Hgb (g/dl)	<*0.0001*	3	5.6	Separate RI
PCV (%)	*0.0033*	3	3.4	Separate RI
MCH (pg)	*0.0016*	3	3.1	Separate RI
MCHC (g/dl)	<*0.0001*	3	5.0	Separate RI
MCV (fl)	0.2929	3	0.3	No separate RI
RDW	0.9577	3	0.6	No separate RI
ESR (mm/h)	0.3283	3	0.9	No separate RI

Z^†^ = critical value, Z* = calculated value, Separate RI are needed only when Z^†^ is greater than Z*, P value is derived from Mann–Whitney U test

Italic values indicate hematological parameters which need partition between male and female (Z^†^ > Z*)

**Table 3:** Median, IQR, range and 95th percentile of reference interval, P values of hematological parameters for adult in Gondar, northwestern Ethiopia

Parameters	N	Median (IQR)	Range (min–max)	RI (95th percentile)	Lower limit 90% CI	Upper limit 90% CI
WBC (×10^9^/l)	240	5.1 (4.2–6.3)	2.7–9.5	3.2–8.8	3.2 (2.9–3.5)	8.8 (8.3–9.1)
Neutrophil (%)	240	53 (48–58)	30–77	36–69	36 (33.8–39.1)	69 (66.9–76.5)
Neutrophils absolute (×10^9^/l)	240	2.7 (2.1–3.5)	1.3–7.3	1.6–5.1	1.6 (1.3–1.7)	5.1 (4.6–6.2)
Lymphocyte (%)	240	38 (33–42)	17–56	22–55	22 (18.2–24.9)	55 (52.4–55.9)
Lymphocytes absolute (×109/l)	240	1.9 (1.5–2.3)	0.70–5.0	1–3.5	1 (0.8–1.1)	3.5 (3.2–4.2)
Mixed (%)	240	9 (8–11)	4–18	6–13	6 (5.4–6.4)	13 (13.0–18.0)
Mixed absolute (×10^9^/l)	240	0.50 (0.4–0.6)	0.2–1.2	0.2–1	0.2 (0.2–0.3)	1.0 (0.9–1.2)
MCV (fl)	240	92 (90–94)	78–105	85–100	85 (80.3–85.1)	100 (99–102)
Platelet (×10^9^/l)	240	264 (210–312)	110–531	128–432	128 (115–140)	432 (403–496)
ESR (mm/h)	240	5.0 (0–5)	0–20	0–15	0 (0–0)	15 (15–20)
RDW	240	14 (13–14)	12–18	12–17	12 (12–12.7)	17 (15.5–18.3)
RBC (×10^12^/l)						
Male	120	5.01 (4.56–5.59)	3.12–6.95	3.53–6.93	3.53 (3.12–3.9)	6.93 (6.45–6.95)
Female	120	4.8 (4.2–5.25)	3.0–6.50	3.45–6.25	3.45 (3.0–3.57)	6.25 (6.08–6.5)
Hgb (g/dl)						
Male	120	14.2 (13.1–15.5)	11.0–19.1	11.5–18.0	11.5 (11–11.9)	18.0 (17.9–19.1)
Female	120	12.9 (12.0–14.3)	10.9–16.9	11.0–16.7	11.0 (10.9–11.3)	16.7 (16.2–16.9)
PCV (%)						
Male	120	46.9 (43.2–50.0)	34.5–60.2	36.2–58.6	36.2 (34.5–37.6)	58.6 (57.0–60.2)
Female	120	45.2 (40.4–48)	30.8–59.0	32.1–56.6	32.1 (30.8–36)	56.6 (52.1–59)
MCH (pg)						
Male	120	29.0 (28–30)	26–34.0	26.6–33.3	26.6 (25.7–27.1)	33.3 (32–34)
Female	120	28.6 (27.7–29.2)	25.6–33.1	25.8–32.8	25.8 (25.6–26.4)	32.8 (31–33.1)
MCHC (g/dl)						
Male	120	31.3 (30.6–32.4)	28.0–36.5	29.5–34.4	29.5 (28–30)	34.4 (33.6–36.5)
Female	120	30.8 (30.0–31.2)	28.0–34.7	28.5–34.4	28.5 (28–29)	34.4 (32.5–34.7)

**Fig. 1:**
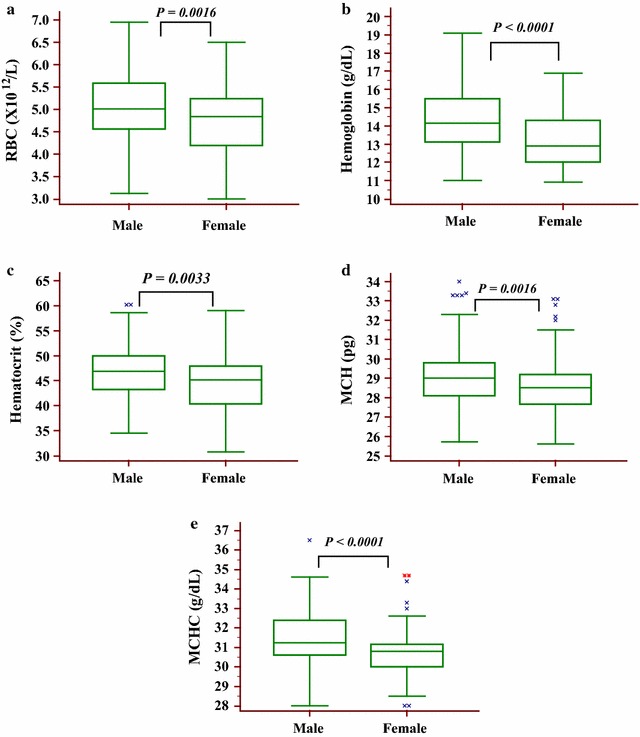
*Box* and *Whisker plots* indicating the effect of sex on hematological parameters of the study participants. **a** Red blood cells, **b** Hemoglobin, **c** Hematocrit, **d** Mean corpuscular hemoglobin, **e** Mean corpuscular hemoglobin concentration

Box and Whisker plots indicate the effect of sex on hematological parameters of the study participants (Fig. 1). The median and 95th percentile reference intervals for RBC, Hgb, PCV, MCH and MCHC were 5.01 (3.53–6.93 × 10^12^/l), 46.9 (36.2–58.6%), 14.2 (11.5–18 g/dl) and 31.3 (29.5–34.4 g/dl) respectively for males and 4.8 (3.45–6.25 × 10^12^/l), 45.2 (32.1–56.6%), 12.9 (11–16 g/dl) and 30.8 (28.5–34.4 g/dl) respectively for females (Table 3).

## Discussion

This study showed a significant gender differences for the RBC parameters (RBC, Hgb, PCV, MCH and MCHC). The finding agrees with the well-established fact that males have higher values than female. While there was no significant differences between genders with regard to WBC parameters, platelet counts, ESR, MCV and RDW. This was also in agreement with previously carried out studies in Addis Ababa and other African countries [1, 6, 15, 17–19] (Tables 4, 5 and 6). The significant difference between male and female may be due to biological and physiological factors such as the influence of the hormone androgen on erythropoiesis and also due to menstrual blood loss in females

**Table 4:** Comparison of median, 95% reference range and Mean ± SD of hematological parameters in this study and others

Parameter	Gondar (current study)			Addis Ababa [17]		Kenya [6]		Burkina Faso (22)		Wintrobe [2]	
	Sex	Median (95% range)	Mean ± SD	Median (95% range)	Mean ± SD	Median (95% range)	Mean ± SD	Median (95% range)	Mean ± SD	Median (95% range)	Mean ± SD
WBC (×10^9^)	Female	5.1 (3.2–8.8)	5.3 ± 1.4	5.9 (3–12.2)	6.2 ± 2.2	4.9 (3.0–9.1)	NA	5.2 (3.4–7.4)	NA	NA (4.3–10)	7.2 ± NA
	Male			5.9 (3–9.8)	6.0 ± 1.8	4.3 (2.7–7.5)	NA	5.1 (3.2–9.2)	NA	NA (4.3–100)	7.2 ± NA
Platelet (×10^9^)	Female	264 (128–432)	267 ± 76	193 (98–352)	202 ± 67	251 (124–444)	NA	252 (159–356)	NA	NA (150–450)	NA
	Male			203 (97–324)	207 ± 62	218 (115–366)	NA	217 (127–365)	NA	NA (150–450)	NA
Neutrophil (absolute) (×10^9^)	Female	2.7 (1.6–5.1)	2.8 ± 0.9	NA	NA	1.96 (0.99–5.6)	NA	NA	NA	NA	NA
	Male			NA	NA	1.78 (0.88–4.3)	NA	NA	NA	NA	NA
Neutrophil (%)	Female	53 (36–69)	53 ± 8	NA	NA	42 (20–70)	NA	46 (30–62)	NA	NA	NA
	Male			NA	NA	42 (20–70)	NA	43 (27–64)	NA	NA	NA
Lymphocyte (absolute) (×10^9^)	Female	1.9 (1–3.5)	2 ± 0.7	1.7 (1.09–3.5)	NA	2.16 (1.29–4.0)	NA	2.2 (1.4–3.2)	NA	NA	NA
	Male			(0.96–3.5)	NA	1.86 (1.12–3.2)	NA	2.1 (1.3–4.0)	NA	NA	NA
Lymphocyte (%)	Female	38 (22–55)	37 ± 8	(17–59)	36 ± NA	45 (20–60)	NA	43 (27–57)	NA	NA	NA
	Male			(17–59)	36 ± NA	45 (20–60)	NA	44 (26–60)	NA	NA	NA
Mix cells (absolute) (×109)	Female	0.5 (0.2–1)	0.5 ± 0.2	NA	NA	NA	NA	NA	NA	NA	NA
	Male			NA	NA	NA	NA	NA	NA	NA	NA
Mixed cells (%)	Female	9 (6–13)	10 ± 2	NA	NA	NA	NA	NA	NA	NA	NA
	Male			NA	NA	NA	NA	NA	NA	NA	NA

*WBC* white blood cells, *SD* standard deviation, *NA* not applicable

**Table 5:** Comparison of median, 95% reference range and Mean ± SD of hematological parameters in this study and others

Parameter	Gondar (current study)		Addis Ababa [17]		Kenya [6]		Burkina Faso (22)		Wintrobe [2]	
	Median (95% range)	Mean ± SD	Median (95% range)	Mean ± SD	Median (95% range)	Mean ± SD	Median (95% range)	Mean ± SD	Median (95% range)	Mean ± SD
RBC (×10^12^)										
Female	4.8 (3.45–6.25)	4.7 ± 0.725	4.5 (3.7–5.2)	4.5 ± 0.4	4.8 (3.7–5.6)	NA	4.2 (3.5–4.9)	NA	NA (4.2–5.5)	4.8 ± NA
Male	5.01 (3.53-6.93)	0.09 ± 0.79	5 (4.3–5.9)	5.1 ± 0.4	5.3 (4.4–6.3)	NA	4.7 (3.8–5.6)	NA	NA (4.5–6.3)	5.4 ± NA
PCV (%)										
Female	45.2 (32.1–56.6)	44.2 ± 5.84	42.1 (35.3–48.8)	42 ± 3.2	40 (30–50)	NA	35 (30–39)	NA	NA (37–47)	42 ± NA
Male	46.9 (36.2–58.6)	6.8 ± 5.8	48.2 (41.6–55.11)	48.3 ± 3.7	47 (40–50)	NA	39 (34–46)	NA	NA (41–51)	46 ± NA
Hgb (g/dl)										
Female	12.9 (11–16.7)	13.2 ± 1.6	14.9 (12.2–16.6)	14.3 ± 3.4	8.44 (5.9–10)	NA	12 (9.8–13.5)	NA	NA (12–16)	14 ± NA
Male	14.2 (11.5–18)	14.4 ± 1.8	16.1 (13.9–18.3)	16.1 ± 1.1	9.9 (8.3–11.3)	NA	13.5 (11.3–15.6)	NA	NA (14–18)	16 ± NA
MCV (fl**)**										
Female		92 ± 3.7	NA	NA	84.9 (66–95.7)	NA	84 (75–92)	NA	NA	NA
Male	92 (85 –100)		NA	NA	88 (71.4–98.2)	NA	85 (73–94)	NA	NA	NA
MCH (pg)										
Female	28.6 (25.8–32.8)	28.5 ± 1.46	NA	NA	NA	NA	28 (24–32)	NA	NA	NA
Male	29 (26.6–33.3)	29.1 ± 1.54	NA	NA	NA	NA	29 (25–34)	NA	NA	NA
MCHC (g/dl)										
Female	30.8 (28.5–34.4)	30.6 ± 1.2	NA	NA	33.9 (32.2–35.2)	NA	34.2 (31.4–36.1)	NA	NA	NA
Male	31.3 (29.5–34.4)	31.5 ± 1.3	NA	NA	34.2 (32.4–35.3)	NA	34.6 (31.7–36.3)	NA	NA	NA

**Table 6:** Comparisons of median and 95% reference range with Eastern and Southern Africa (18), Tanzania (19), Togo (20) and Uganda (21) of African countries

Parameters	Gondar	Togo	Tanzania	Uganda	Eastern and southern Africa
	Median (95% range)	Median (95% range)	Median (95% range)	Median (95% range)	Median (95% range)
WBC (×10^9^/l)	5.1 (3.2–8.8)	4.1 (1.9–10.1)	4.6 (3.0–7.9)	4.9 (2.8–8.2)	3.1–9.1
Neutrophils (Abs)	2.7 (1.6–5.1)	1.6 (0.5–5.4)	2.2 (1.1–4.7)	NA	1.0–5.3
Neutrophils (%)	53 (36–69)	NA	48.1 (32.0–69.1)	38.9 (22.2–59.3)	25–66
Lymphocyte (Abs)	1.9 (1–3.5)	2.1 (1.1–4.3)	1.8 (1.1–3.0)	NA	1.2–3.7
Lymphocyte (%)	38 (22–55)	NA	40.5 (20.8–56.7)	44.4 (26.7–61.2)	23–59
Mixed (Abs)	0.5 (0.2–1)	NA	0.5 (0.3–1.1)	NA	NA
Mixed (%)	9 (6–13)	NA	10.3 (5.6–19.8)	NA	NA
Platelet (×10^9^/l)	264 (128–432)	239 (120–443)	244 (150–398)	218.5 (109–384)	126–438
MCV (fl)	92 (85–100)	84.5 (80–99)	89.4 (77.6–98.1)	85 (71–97)	68–98
RBC (×10^12^/l)					
Male	4.8 (3.45–6.25) 5.0	5.0 (3.3–6.4)	5.21 (4.41–6.27)	5.0 (3.8–6.1)	4.0–6.4
Female	1 (3.53–6.93)	4.5 (3.1–6.0)	4.69 (3.84–5.59)	4.4 (3.3–5.3)	3.8–5.6
Hgb (g/dl)					
Male	12.9 (11–16.7)	15.8 (10.0–18.4)	15.4 (13.7–17.7)	14.5 (11.6–17.1)	12.2–17.7
Female	14.2 (11.5–18)	13.0 (10.3–17.1)	13.5 (11.1–15.7)	12.8 (9.8–16.2)	9.5–15.8
PCV (%)					
Male	45.2 (32.1–56.6)	42.8 (28.0–54.0)	46.6 (40.2–53.7)	42.6 (33.8–49.5)	35.0–50.8
Female	46.9 (36.2–58.6)	38.1 (28.0–47.0)	41.5 (36.2–46.8)	37.8 (28.3–46.8)	29.4–45.4
MCH (pg)					
Male	28.6 (25.8–32.8)	29.7 (26–36)	30.0 (23.1–33.2)	29.2 (23.0–33.8)	NA
Female	29 (26.6–33.3)	29.3 (25–37)	29.3 (24.2–33.1)	29.5 (24.8–32.7)	NA
MCHC (%)					
Male	30.8 (28.5–34.4)	35.1 (29–39)	33.3 (30.6–35.1)	34.2 (32.4–35.3)	NA
Female	31.3 (29.5–34.4)	35.1 (30–41)	32.7 (30.4–34.8)	34.2 (33.0–35.5)	NA
RDW (%)					
Male	14 (12–17)	NA	NA	12.8 (10.9–16.8)	NA
Female	14 (12–17)	NA	NA	12.7 (11.0–17.3)	NA

*Abs* absolute

The 95th percentile reference interval of the current study lower limit of RBC in both sexes, were lower than studies conducted in Addis Ababa Ethiopia, Kenya, Burkina Faso and text books while the upper limit was higher than the above mentioned studies (Tables 4, 5 and 6). Comparison of hematological results with this study indicated that similarity could not be found based on geographical proximity, this may be due to difference in altitude, method and instrument used for analysis [6, 8, 15, 18].

The lower limit of hemoglobin reference interval in this study was lower than a study conducted in Addis Ababa and text books while higher than studies conducted in Kenya and Burkina Faso for both genders. On the other hand the upper limit of Hgb was higher than Addis Ababa, text books, Kenya, and Burkina Faso. This difference may be due to altitude and ethnic variations. Thus, it may not be possible, even, to have one standard reference between two localities in the same county as experienced in Kenya. Similarly the lower limit reference intervals of PCV in both sexes was lower than study conducted in Addis Ababa, text books and higher than studies conducted in Kenya and Burkina Faso. While the upper limit was higher than studies carried out in Addis Ababa, Kenya, Burkina Faso and text books [6, 8, 15, 18]. Likewise of RBC and Hgb the difference in the reference interval values might arise from differences in altitude and ethnical variations.

The current study reference interval values of MCV are generally higher than values indicated in different African countries. The lower limit values of MCH in both sexes in this study was higher than a study conducted in Burkina Faso, but the upper limit was comparable, but the established values of MCHC in both sexes was generally lower than values indicated in different African countries [6, 18]. The WBCs reference intervals established for the general population in the current study was lower than the values described for Caucasian population in different text books, Addis Ababa, Burkina Faso and Kenya. However, median and 95 percentile ranges of neutrophils and lymphocytes of this study compared with similar study conducted on Caucasian, African, Afro-Caribbean and Jamaican population, neutrophils were lower than Caucasian and Afro-Caribbean, but higher than African and Jamaica. Lymphocyte count in the current study was higher than Caucasian and African black population [6, 8, 15, 18]. The difference between the current result and other findings might be due to geographical differences, environment, diet, and ethnic background [16].

The median and 95th percentile range values of ESR was at normal range with no difference for male and female as all the recruited subjects were healthy, not exposed to inflammatory conditions associated with systemic inflammatory diseases [20].

## Conclusions

Haematological reference intervals established from adult healthy population of Northwest Ethiopia (Gondar and surrounding areas) were different from Caucasian and African counties. The haematological intervals were also different from previous results obtained in other part of Ethiopia. Intervals for red blood cell count, hematocrit values, hemoglobin concentration, MCH and MCHC were different for male and female but there was no difference in WBC parameters, PLT, MCV, RDW and ESR.Further studies on haematological intervals for all age groups are recommended for locally derived standard haematological intervals (Additional file 1).

## Additional file

**Additional file 1.** Questionnaire for data collection of a study on Hematological reference intervals determination in adults at Gondar university hospital, Northwest Ethiopia.

## Abbreviations

AIDS: acquired immune deficiency syndrome
BMI: body mass index
CLSI: Clinical Laboratory Standard Institution
EDTA: ethylene diamine tetra-acetic acid
HbsAgs: hepatitis B virus surface antigen
HcAbs: hepatitis C virus antibodies
Hgb: hemoglobin
HIV: human immunodeficiency virus
IFCC: International Federation of Clinical Chemistry
LPT: lymphocyte
MCH: mean corpuscular hemoglobin
MCHC: mean corpuscular hemoglobin concentration
MCV: mean corpuscular volume
NCCLS: National Committee for Clinical Laboratory Standards
NTL: neutrophils
PCV: packed cell volume
PLT: platelet
RBC: red blood cells
RDW: red cell distribution width
RIs: reference intervals
WBC: white blood cells
