# Supplementary material for: Hematological reference intervals determination in adults at Gondar university hospital, Northwest Ethiopia
Source: BMC Res Notes. 2016 Nov 2;9:483. doi: 10.1186/s13104-016-2288-8 (PMC5093958; doi:10.1186/s13104-016-2288-8)
Supplement: Supplementary file 1 — Additional file 1. Questionnaire for data collection of a study on Hematological reference intervals determination in adults at Gondar university hospital, Northwest Ethiopia. [file 13104_2016_2288_MOESM1_ESM.docx]

**Questionnaire for data collection of a study on Hematological reference intervals determination in adults at Gondar university hospital, Northwest Ethiopia**

**Code number_________________**

| **Questionnaire used to collect socio-demographic data** | | | | |
| --- | --- | --- | --- | --- |
| **Sr. No** | **Question** | **Response** | | |
| 101 | Age in year | ________________ | | |
| 102 | Sex | 1. Male 2. Female | | |
| 103 | Weight in Kg.  Height in meter and centimeter | _________________  ________________ | | |
| 104 | Address | 1. Gondar 2. Outside Gondar | | |
| 105 | Marital status | 1. Married 2. Single 3. Divorced 4. Widowed | | |
| 106 | Religion | 1. Orthodox 2. Muslim 3. Protestant 4. Other specify | | |
| 107 | Educational status | 1. Unable to read 2. Read and write 3. Primary education 4. Secondary education (9-12) and above | | |
| 108 | Occupation | 1. Private 2. Government employees 3. Farmer 4. House wife | | |
| **Questionnaire to collect history and clinical data (As exclusion criteria)** | | | | |
| **Sr. No** | **Questions** | | ***Response*** | |
|  |  |  | ***Yes*** | ***No*** |
| 201 | Do you consider that yourself to be healthy? | |  |  |
| 202 | Have you been sick recently?  if yes, when________________ what____*___________* | |  |  |
| 203 | Are you taking prescribed medication?  If yes, what?_________________ | |  |  |
| 204 | Do you have high blood pressure | |  |  |
| 205 | Are you exposed in hazardous chemicals in your job?  If yes, what | |  |  |
| 206 | Do you drink alcohol beverage?  If yes what form | |  |  |
| 207 | Are you currently under doctor care?  If yes, what ***________________*** | |  |  |
| 208 | Is there any inherited health disorder in your family?  If yes, describe | |  |  |
| 209 | Have you taken any pain relievers recently?  If yes, what________________when | |  |  |
| 210 | Do you have tuberculosis? | |  |  |
| 211 | Do you have lymphadenopathy? | |  |  |
| 212 | Do you lose your weight?  If yes how much? (Kg) | |  |  |
| 213 | Do you exercise regularly?  If yes how often (per week)_____________________ | |  |  |
| 214 | Are you using tobacco (smoking)? | |  |  |
| 215 | Do you taken supplementary vitamins?  If yes what? _____________________________ | |  |  |
| 216 | Have you take’ any cold or allergy medication recently?  If yes what? _________________________________ | |  |  |
| 217 | Have you taken any anti-acid medication Recently?  If yes what? _________________________________ | |  |  |
| 218 | Do you have fever?  If yes how long specify____________________________ | |  |  |
| 219 | Do you have malaria? | |  |  |
|  | **Females only can be asked the following questions** | | | |
| 220 | Are you still menstruating? | |  |  |
| 221 | Are you pregnant? | |  |  |
| 222 | Are you using oral or implant contraceptive? | |  |  |
